# Big picture vs. small details: exploring how front-of-package labels influence healthy food consumption and cognitive processing

**DOI:** 10.3389/fnut.2026.1881270

**Published:** 2026-08-14

**Authors:** Yongkang Hou, Aocheng Zhou, Junqing Xu, Shaopeng Che

**Affiliations:** 1 School of Journalism and Communication, Tsinghua University, Beijing, China; 2 Center for Japanese Studies, Shanghai Jiao Tong University, Shanghai, China; 3 School of Humanities and Social Science, Chang’an University, Xi’an, China

**Keywords:** cognitive processing experiment, front-of-pack label, health food consumption, health perception, nutrition accuracy

## Abstract

**Background:**

Front-of-package labeling (FoPL) has been increasingly implemented as a public health nutrition strategy to promote healthier dietary choices and reduce the burden of diet-related chronic diseases. However, the effectiveness of different FoPL formats and the cognitive pathways associated with consumers’ food perceptions and choices remain unclear. This study aimed to examine how different FoPL types influence consumers’ health perceptions, food choices, and cognitive pathway patterns.

**Methods:**

Two experimental studies were conducted. Study 1 employed a 2 × 2 between-subjects online experiment comparing FoPL type (summary vs. nutrient-specific) and color coding (absent vs. present) among 237 participants. Two-way ANCOVA was used to examine the effects of FoPL design on health perception changes and food choice changes while controlling for health food interest and functional health literacy. Study 2 expanded the stimuli to multiple low-health food categories and compared three experimental conditions among 138 participants: a Health Star Rating summary label, a Multiple Traffic Light-style color-coded nutrient-specific label, and a No-FoPL control condition. Partial least squares structural equation modeling (PLS-SEM) was used to examine indirect associations involving nutrition accuracy, food health perception, and intentions to purchase low-health foods.

**Results:**

In Study 1, a significant interaction effect between FoPL type and color coding was observed for health perception changes (*F* = 11.08, *p* < 0.001). Specifically, nutrient-specific labels with color coding generated significantly higher perceived healthfulness than nutrient-specific labels without color coding. However, no significant main or interaction effects were found for food choice changes. In Study 2, the summary-label condition showed a significant indirect association with lower intentions to purchase low-health foods through food health perception (β = −0.326, *p* = 0.028). In contrast, the color-coded nutrient-specific-label condition showed a significant sequential indirect association involving nutrition accuracy and food health perception (β = −0.119, *p* = 0.044). The two models explained substantial variance in intentions to purchase low-health foods, with R^2^ values of 0.504 for the summary-label model and 0.404 for the nutrient-specific-label model.

**Conclusion:**

Different FoPL designs were associated with distinct cognitive pathway patterns. Summary labels were more closely linked to food health perception, whereas nutrient-specific labels, especially when combined with color coding, were linked to nutrition accuracy and subsequent health perception. These findings provide practical implications for optimizing FoPL policies and designing more effective nutrition communication strategies to promote healthier dietary behaviors.

## 1 Introduction

The widespread availability and overconsumption of processed foods are key drivers of nutrition-related chronic diseases (1). Unhealthy diets and high energy intake significantly contribute to the global disease burden, leading to 11 million deaths and severe health consequences for 255 million people worldwide (2). In retail food environments, although Nutrition Facts Panels (NFP), typically displayed on the back of food packages, are widely mandated and provide detailed nutrient information, their effectiveness in promoting healthier food consumption remains limited (3). Consumers rarely consult NFPs during food selection (4, 5), partly because interpreting detailed nutrient information requires sufficient nutritional knowledge, motivation, and time (6–8).

Front-of-package labeling (FoPL) has therefore been proposed as a more visible and accessible strategy for improving consumer understanding and promoting healthier food choices. FoPL systems place simplified nutrition information on the front of packages and use graphic symbols, summary indicators, or color-coded cues to communicate product healthfulness in a more salient and interpretable way (8). Compared with traditional NFPs, FoPLs provide simplified, interpretive, and highly visible nutrition information that can help consumers identify healthier products in complex food environments (9–11). The World Health Organization has recognized FoPL as a “best-buy” intervention for addressing non-communicable diseases (12), and by 2024, FoPL systems had been implemented in more than 44 countries (13).

However, FoPL systems remain diverse in format and vary in effectiveness, with no consensus on the “best” approach. One reason for this variation is that different FoPL formats do not present the same type of nutrition information. Rather, they differ in their information content and structure (14). Nutrient-specific labels, such as the UK’s Multiple Traffic Light (MTL),^1^ preserve nutrient-level details. Summary labels, such as Australia’s Health Star Rating (HSR)^2^, compress multiple nutritional attributes into an overall evaluative score (14). Prior studies suggest that both formats can improve perceived healthfulness and support healthier choices compared with NFPs (3, 11, 15). However, their relative advantages remain debated. Nutrient-specific labels may support more accurate interpretation of nutrient information, whereas summary labels may reduce cognitive burden and facilitate rapid judgment (16–19). Therefore, the key issue is not only whether one label format is more effective than another, but also how different information structures guide consumers’ processing of nutrition information.

Color coding is closely related to this information-processing issue. Color-coded FoPLs have been discussed as a public health communication strategy because they can make nutrition information more salient and easier to interpret, thereby nudging consumers toward healthier products (20). The logic model of color-coded labels suggests that FoPL effects may unfold through earlier cognitive responses, such as understanding and evaluating nutrition information, before influencing downstream purchase-related outcomes (20). Therefore, color coding should not be understood merely as a decorative visual feature or as a cue that produces uniform effects across all labels. Its role may depend on the information structure in which it is embedded.

However, existing research has not sufficiently clarified this conditional role of color coding. Many studies compare complete FoPL systems without separating color cues from label format, making it difficult to determine whether observed effects are driven by information structure, color coding, or their combination (14). Other studies examine color within a single FoPL format, which limits understanding of whether color functions similarly across different label structures (21). Some international findings suggest that color-coded summary labels may outperform more detailed monochrome formats in certain contexts, indicating that color can enhance the communicative value of FoPLs (22). Yet these findings also highlight the need to examine color coding together with label information structure rather than treating it as an isolated visual feature. As a result, less is known about how FoPL type and color coding jointly shape consumers’ processing of nutrition information (17).

Beyond label format and color coding, another important question concerns how consumers process the information provided by different FoPL designs. Existing research suggests that FoPLs may influence food-related responses through at least two proximal cognitive routes: improving the accuracy of nutritional understanding (16) and shaping overall health perceptions of the product (23, 24). These two routes are related but conceptually distinct. Nutrient-specific labels require consumers to identify and interpret information about individual nutrients, such as fat, sugar, sodium, or saturated fat, making nutrition accuracy particularly relevant to this form of label processing. Summary labels, by contrast, provide a more aggregated evaluation of product healthfulness, making food health perception a more direct cognitive response to this type of information structure. However, existing research has not sufficiently clarified whether summary and nutrient-specific labels guide consumers through similar or different cognitive routes before shaping decision-related responses.

Based on this logic, the present study examines how FoPL type and color-coding strategy influence consumers’ health perceptions and food consumption decisions, and further explores whether policy-relevant FoPL configurations are associated with different cognitive pathway patterns. Two complementary online experiments were conducted. Study 1 used a 2 (FoPL type: summary vs. nutrient-specific) × 2 (color coding: absent vs. present) between-subjects design to examine how label format and color coding independently and jointly influence health perception changes and immediate food choice changes. Study 2 then moved from a factorial test of design features to a comparison of two policy-relevant FoPL configurations: an Australia’s HSR (summary label without color coding) and UK’s MTL (nutrient-specific label with color coding). Study 2 examined whether these two real-world label configurations were associated with different indirect pathway patterns involving nutrition accuracy, food health perception, and intentions to purchase low-health foods. This study contributes to the FoPL effectiveness literature by disentangling label format from color coding and testing their interaction. It also advances the information-processing perspective on FoPL by examining observable cognitive pathways involving nutrition accuracy and health perception. The findings offer practical implications for policymakers, food companies, and retailers by clarifying when simplified summary labels and color-coded nutrient-specific labels may be more useful for guiding healthier consumer decisions.

## 2 Theoretical background and research questions

### 2.1 FoPL effectiveness: information structure and healthy responses

A key theoretical distinction among FoPL systems lies in how nutritional information is structured and communicated. Previous research distinguishes between two primary formats: nutrient-specific labels, which provide evaluative cues for individual nutrients (e.g., MTL), and summary labels, which offer an overall assessment of a product’s nutritional quality (e.g., HSR) (14). Both formats have generally shown more positive effects than traditional NFPs, but their cognitive and behavioral impacts may differ across contexts (15, 25–28).

Nutrient-specific labels may improve consumer decision-making by making nutrients of concern more visible and interpretable. By directing attention to individual nutrient levels, such labels can help consumers better recognize health-related factors and make healthier choices (17). Recent evidence also suggests that nutrient-specific labels (e.g., MTL) can significantly influence food choices compared with other label formats, including warning labels and simplified labels (29). From this perspective, nutrient-specific labels may be especially useful when consumers need to evaluate the nutritional quality of a product in a more precise and differentiated way (16).

Summary labels, by contrast, reduce complex nutritional information into an overall evaluative signal. This simplified structure may allow consumers to make quick judgments about product healthfulness based on accessible criteria, such as whether a product appears generally “good” or “bad.” Prior studies have found that consumers often perceive summary labels as easier to understand, which may support healthier decision-making (19, 25). For example, the study found that summary labels (e.g., HSR) were more effective than nutrient-specific labels (e.g., MTL) in discouraging unhealthy purchases (19), while the other study showed that both labels improved healthfulness perceptions and healthier choices (7), with summary labels showing a slight advantage across many product categories.

These findings point to a trade-off between informational detail and cognitive simplicity. Nutrient-specific labels provide more detailed information and may support accurate nutrient-level interpretation, but they may also increase cognitive load because consumers must process and integrate multiple indicators simultaneously (30). In contrast, summary labels reduce cognitive effort and facilitate rapid evaluation, but they may oversimplify nutritional information and produce a “health halo” effect when consumers rely too heavily on the overall score while overlooking unfavorable nutrient levels (31). Both mechanisms may affect consumers’ health perceptions and food choices in different ways. Given these mixed findings, this study poses the following research questions:

*RQ1*: How do summary and nutrient-specific FoPLs differ in promoting consumers’ perceptions of food healthiness (a) and healthy food choices (b)?

### 2.2 Colored-coding strategy as an interpretive cue

Color coding is widely used in nutrition and health-related labels because colors can make information more salient and easier to interpret. The use of color in nutrition labels carries the expectation that visual cues may facilitate the processing of nutritional information, leading to more accurate perceptions of food healthiness and potentially healthier food choices (32). Colors carry symbolic meaning with psychological implications (33). According to color-in-context theory, the meaning and effect of color depend on the context in which it is encountered (34). In food labeling contexts, traffic-light colors are particularly meaningful because green, amber, and red are commonly associated with “go,” “caution,” and “stop,” respectively. Therefore, color cues in FoPLs may help consumers attach evaluative meaning to nutrition information and judge food healthfulness more efficiently (35). The study suggest that color cues and nutrient content claims can influence consumers’ healthfulness perceptions and purchase intentions (36), and FoPLs incorporating color are less likely to be overlooked than monochrome labels (6). Recent reviews also suggest that color-coded labels can guide consumers toward healthier choices (37).

However, the effect of color coding should not be understood as a universal main effect that operates in the same way across all FoPL formats. Existing empirical evidence suggests that the role of color coding is conditional and context-dependent.

The studies find that color coding may primarily improve the accurate interpretation of nutrition information and may not consistently translate into healthier choices across all contexts (32). Cross-national evidence further shows that color-coded summary labels, such as Nutri-Score or simplified color-coded summary systems, may outperform more detailed monochrome labels in some tasks, whereas in some countries, including Canada and China, monochrome labels or labels providing specific nutrient information may perform better (22). These mixed findings suggest that the effect of color coding depends not only on the presence of color itself, but also on the type of nutrition information with which color is combined.

Based on this reasoning, this study asks:

*RQ2*: How does color coding interact with FoPL type in promoting consumers’ perceptions of food healthiness (a) and healthy food choices (b)?

### 2.3 Potential indicators: functional health literacy and health food interest

Consumer individual variables may influence the overall effectiveness of FoPLs (6, 38–41). One factor that can affect an individual’s ability to interpret nutrition labels and adopt health-related behaviors is functional health literacy (7, 42), which is defined as the capacity to evaluate, understand, and access health and nutritional information. Low functional health literacy has been recognized as an impediment to individual calculation, comprehension, and interpretation of nutrition-related information (43). Malloy-Weir and Cooper found that regardless of the type of FoPL used, individuals with low health literacy were more likely to incorrectly perceive the healthiness of unhealthy food products and purchase them.

Subjective motivations such as health food interest also stand as crucial factors influencing individual responses to food products. Research has shown that the presence of nutrition and health labels is likely to influence individual responses and acceptance of a food product in ways that align with their health-related attitudes and interests in healthy eating (40, 44). The study indicated that individuals with a high degree of health interest had a greater intention to purchase spreads with health claim labels (45). Additionally, recent evidence shows that individuals with higher nutrition literacy use FoPLs more frequently and accurately, reinforcing the link between literacy and healthier food choices (37).

### 2.4 How FoPLs facilitate healthier food consumption: potential cognitive pathways for nutrition accuracy and health perception

Although many studies have examined whether FoPLs influence food evaluations and choices, less is known about how different FoPL formats work through distinct cognitive pathways. Existing evidence suggests that FoPL effects may be associated with two related but conceptually distinct cognitive responses: accuracy of nutritional understanding (16) and product health perception (23, 24). Nutrition accuracy refers to consumers’ ability to correctly identify and interpret nutrient levels, whereas food health perception reflects their overall evaluation of whether a product is healthy, nutritious, or good for them. Distinguishing these mechanisms is important because different FoPL formats may not simply vary in effectiveness but may guide consumer judgment through different forms of information processing.

From a dual-process and heuristic–systematic perspective, consumers may process nutrition labels through relatively quick, low-effort judgments or through more deliberate, detail-oriented processing (46, 47). Recent research further suggests that FoPL processing involves attention allocation, motivation, and perceptions, and that contextual factors such as knowledge may shape how consumers respond to labels (48). Similarly, eye-tracking evidence indicates that food label claims can function not only as heuristic cues but also as systematic cues when information structures encourage deeper processing (49). These findings suggest that FoPLs may guide food decisions by shaping both rapid evaluative judgments and more detailed nutritional interpretation.

Summary labels, such as HSR, condense multiple nutritional attributes into a single evaluative signal. This simplified design may reduce cognitive effort and help consumers quickly infer whether a product is generally healthy or unhealthy. Therefore, summary labels may primarily affect purchase intentions by shaping overall food health perception. For example, HSR labels have been shown to improve health perceptions of packaged foods and subsequently influence purchase intentions (50). However, because summary labels compress detailed nutrition information, they may have a weaker effect on consumers’ nutrient-level understanding.

Nutrient-specific labels, such as MTL, provide more detailed information about individual nutrients and may therefore support more accurate interpretation of nutritional content. By making unfavorable nutrient levels, such as high sugar, sodium, or saturated fat, more visible and interpretable, nutrient-specific labels can reduce perceived product healthfulness and discourage less healthy choices (16, 23). This mechanism is particularly relevant for low-health products, where improved awareness of unfavorable nutrient profiles may lower health perception and reduce purchase intentions (51). When nutrient-specific labels use color coding, they may also provide rapid interpretive cues, allowing consumers to identify unfavorable nutrient levels with less effort (52, 53).

In the present study, food health perception captures an overall evaluative judgment that may reflect simplified, heuristic-like processing, whereas nutrition accuracy captures more detailed comprehension of nutrient-level information and reflects a more systematic form of label interpretation.

Individual characteristics may further shape these pathways. Functional health literacy is relevant because interpreting nutrition information requires the ability to access, understand, evaluate, and apply health-related information (7, 42). Consumers with lower health literacy may have greater difficulty interpreting nutrient information and may be more likely to misperceive unhealthy products as healthy (43). Health food interest may also influence food-related decision-making because consumers who are more interested in healthy eating are more likely to attend to nutrition information and use it when evaluating products (39, 40).

**FIGURE 1:**
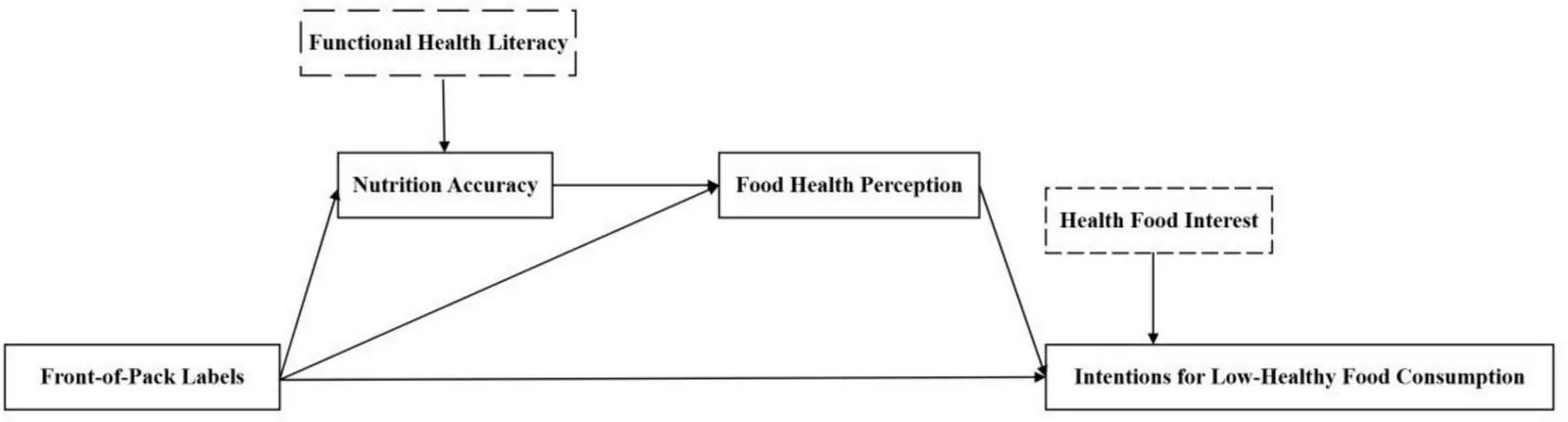
Potential indirect processing model of FoPLs.

In summary, different FoPLs may significantly impact consumers’ intentions for healthy food consumptions by enhancing nutritional accuracy and food health perception. Therefore, this study raises the final research question (an exploratory model illustrating these effects is shown in Figure 1):

*RQ3*: What indirect pathway patterns are associated with the effects of FoPL configurations on consumers’ intentions to purchase low-health foods?

## 3 Materials and methods

This research consisted of two between-subjects experiments designed to examine the effects of different FoPL interventions on consumers’ health perception and food choice, as well as to explore potential cognitive pathway patterns associated with FoPL effects. Study 1 adopted a 2 (FoPL type: summary label vs. nutrient-specific label) × 2 (color coding: absent vs. present) design to examine the independent and interactive effects of label format and color coding on health perception and healthy food choice. Study 2 included a No-FoPL control condition and examined indirect associations linking policy-relevant FoPL configurations, nutrition accuracy, food health perception, and intentions to purchase low-health foods.

Participants in both studies were undergraduate students enrolled at a university in southern China, recruited through convenience sampling. The use of university students is common in behavioral and FoPL intervention research (54), particularly in exploratory or mechanism-testing phases (55). Prior evidence also suggests that university students’ nutrition-related decision-making and label-use behaviors are generally consistent with those of the broader consumer population (56), supporting the suitability of this sample.

The unit of analysis in both experiments was the individual participant. Data were collected through two online experiments conducted in November 2023 (Study 1) and April 2024 (Study 2), respectively. Participants accessed the studies via a Chinese online survey platform.^3^ Before both study procedures commenced, participants were presented with an electronic informed consent form describing the study purpose, procedures, voluntary nature of participation, anonymity, data confidentiality, and their right to withdraw at any time without penalty. Only participants who actively indicated consent by clicking an “Agree” button and were aged 18 years or older were allowed to proceed to the experimental tasks.

All study procedures complied with established ethical standards for social science research and were granted ethics review exemption by the Clinical Research and Applied Ethics Committee of the Third Affiliated Hospital of Guangzhou Medical University. Further information on sampling, procedures, measures, and analytical strategies is provided in the following subsections.

### 3.1 Methods of Study 1

#### 3.1.1 Experimental design, sampling, and procedure

**FIGURE 2:**
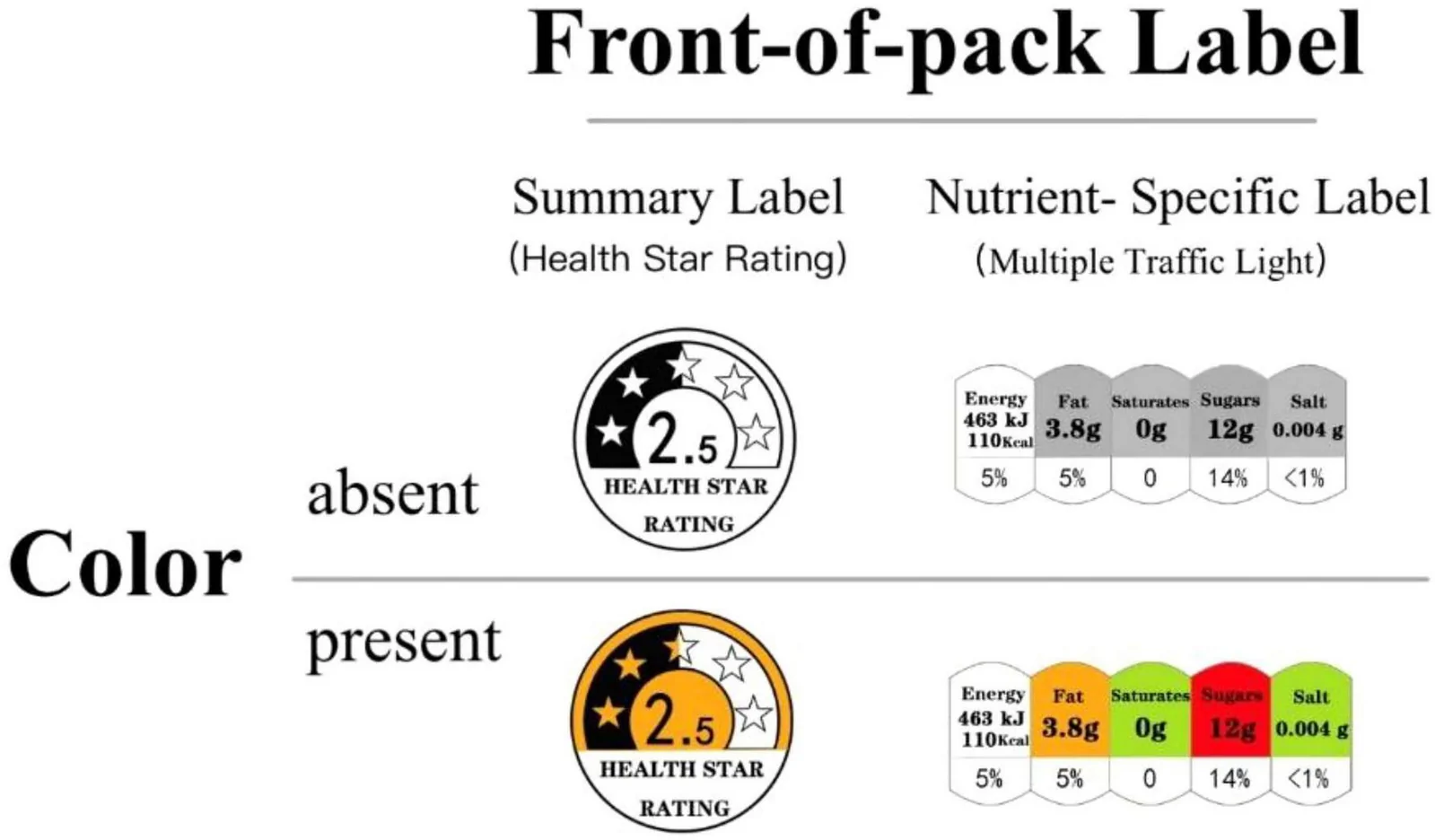
Experimental conditions in study 1.

The design of FoPL styles and nutrient transformations was based on government standards: the HSR system from Australia for summary labels and the MTL system from the UK for nutrient-specific labels. In Study 1, color coding was manipulated as an independent design feature in order to disentangle the effect of label format from the effect of visual color cues. In the absent-color condition, all FoPLs were shown in black and gray. In the present-color condition, color coding followed UK MTL standards. For summary labels, star ratings were color-coded accordingly: 1–2 stars in red, 2.5–3.5 in orange, and 4–5 in green (see Figure 2).

**FIGURE 3:**
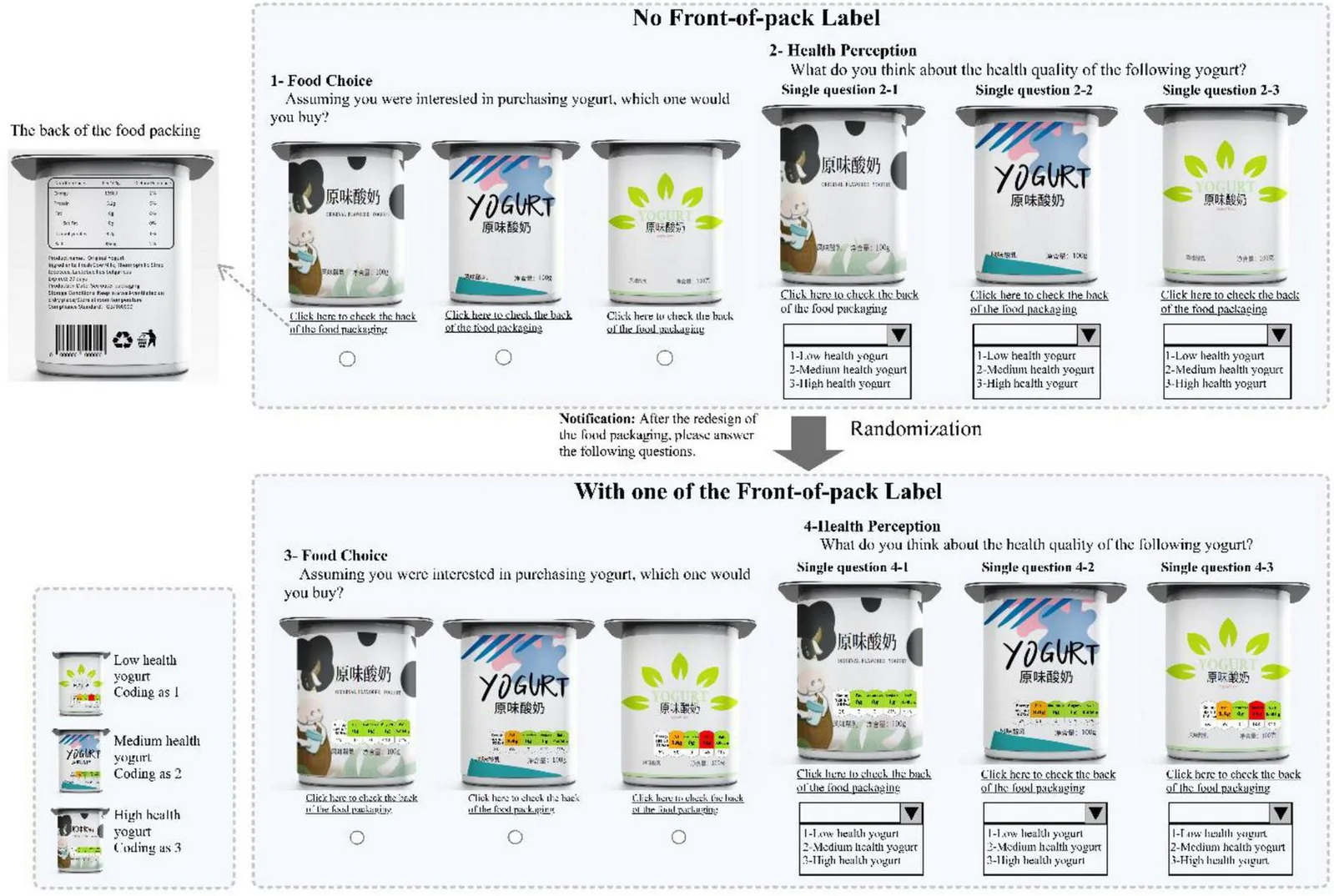
Experimental procedure in study 1.

The main experimental tasks followed the previous study (26) (see Figure 3). Participants first viewed three yogurt products^4^ differing in health quality.^5^ At the same time, participants were asked to make a selection. During this phase, they could click links to view the NFP on the back of the packages, simulating real-life behavior when seeking nutritional information.

Subsequently, participants were informed that the product packaging had been redesigned and were randomly assigned to one of the four FoPL conditions (2 FoPL type × 2 color coding). They then re-evaluated the health quality of the products under the new labeling conditions (see Figure 3). Finally, participants completed questions regarding covariates, demographic information, and whether they had checked the nutritional information on the back of the package.

To ensure data quality, participants who completed the experiment in less than 120 s (*N* = 7) were excluded, resulting in a final valid sample of 237^6^ (see Table 1 for sample description). All experimental materials, including instructions, stimuli, and questionnaires, were presented in Chinese. The translated materials in this study were reviewed by another researcher to ensure accuracy and linguistic consistency.

**TABLE 1:** Sample description and random manipulation.

Variables	Category	N	M/Total score (SD)/percentage	Random test
Sex	Male	60	25.30%	χ^2^(3) = 2.145 *p* = 0.548
	Female	177	74.70%	
Age		237	20.87(1.46)	*F*(3, 236) = 1.62, *p* = 0.186
BMI^1^		237	20.75 (3.9)	*F*(3, 236) = 1.75, *p* = 0.159
Health food interest (HFI)		237	3.09(0.77)	*F*(3, 236) = 1.58, *p* = 0.194
Functional health literacy (FHL)		237	3.51(1.42)	*F*(3, 236) = 1.87, *p* = 0.136

^1^BMI was calculated as weight in kilograms divided by height in meters squared. Participants were categorized as underweight (BMI < 18.5), normal weight (BMI = 18.5—23.9), or overweight (BMI ≥ 24).

#### 3.1.2 Measures

**FIGURE 4:**
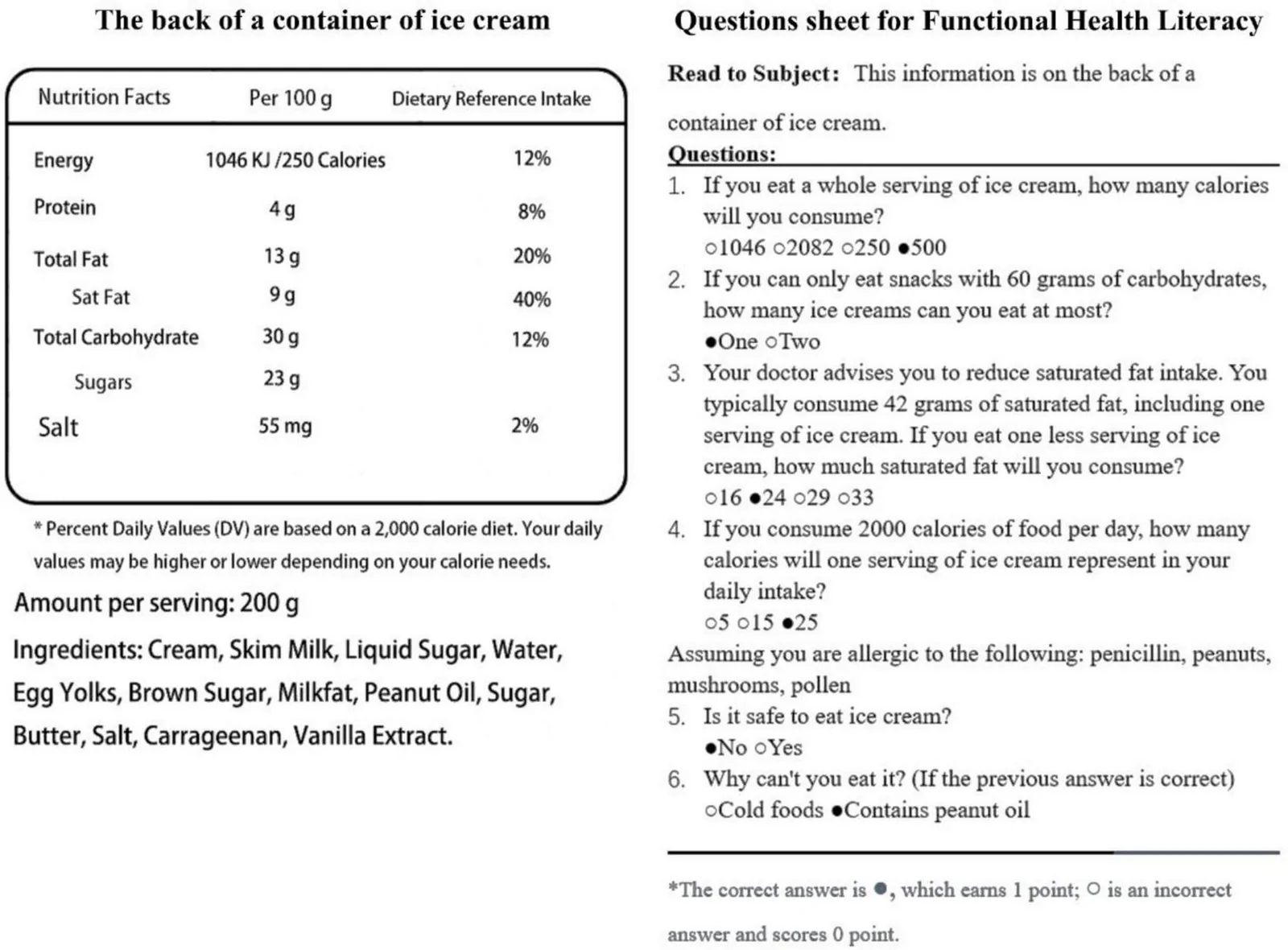
The process and questions of testing functional health literacy.

This study measured *Food Choice Changes* (FCC), *Health Perception Changes* (HPC), *Health Food Interest* (HFI), *Functional Health Literacy* (FHL) (see Figure 4) and demographic variables (details see Table 2).

**TABLE 2:** Measurement details in Study 1.

Measures	Definition	Details	M/SD	α	References
Food choice changes (FCC)	Change in selection of healthier food items due to FoPL exposure	Participants chose among yogurt options with different health levels (High = 3, Medium = 2, Low = 1). FCC is calculated as the difference between choices with and without FoPL (range: −2 to 2) (see Figure 3). A negative score indicates a decline in healthy food selection (misleading effect), 0 indicates no change, and a positive score indicates improvement in healthy choices as influenced by FoPL.	*M* = 0.37, SD = 0.83	–	Egnell et al. (9)
Health perception changes (HPC)	Change in accuracy of healthiness judgments for food items	This measures the difference between actual and perceived health levels. When participants assess the perceived health quality of yogurt with varying levels of healthiness, if the score of actual health quality (High health yogurt = 3, Medium health yogurt = 2, Low health yogurt = 1) aligns with participant perceived health quality (e.g., high-quality yogurt being perceived as high-health yogurt), there is no perceptional error. Perceptual errors (*Error*) occur when there is a difference between the actual health quality and the perceived health quality (S). The algorithms for calculating perceptual errors for yogurts of three different health levels (high, medium, and low), as high-health yogurts are more likely to be underestimated, whereas low-health yogurts tend to be overestimated. Therefore, the calculation methods for perceptual errors at each health level are as follows: *Error*_*Low*_ = 1− S_*low*_ *Error*_*Medium*_ = 2−|S_*medium*_-2| *Error*_*High*_ = S_*low*_–3 The difference between the sum of perceptual errors for high, medium, and low-health foods with FoPL conditions (*Total Error_*Label*_*) and the sum of perceptual errors without FoPL conditions (*Total Error _*without label*_*) is defined as HPC (*Total Error_*Label*_ – Total Error _*without label*_*). Specifically, negative values indicate an overall decrease in perceptual accuracy of health quality, 0 indicates no overall change, and positive values indicate an overall improvement in perceptual accuracy. As the numerical value increases, ranging from −3 to 4, it signifies an enhancement of the accuracy of judgments regarding the overall health quality of food items.	–	–	Egnell et al. (26)
Health food interest (HFI)	Degree of personal interest in eating healthily	Items: I am very particular about the healthiness of food. I always follow a healthy and balanced diet. It is important for me that my diet is low in fat. It is important for me that my daily diet contains a lot of vitamins and minerals. I eat what I like and I do not worry about the healthiness of food (R). The healthiness of food has little impact on my food choices (R). The healthiness of snacks makes no differences to me (R). I do not avoid any foods, even if they may raise my cholesterol (R). (1 = strongly disagree to 5 = strongly agree)	*M* = 3.09, SD = 0.77	0.75	Sabbe et al. (40)
Functional Health Literacy (FHL)	Ability to understand and interpret health and nutrition information, including food labels	The measurement approach was adapted from the *Test of Functional Health Literacy in Adults.* Figure 4 for the process and questions. Higher scores represent greater ability to interpret nutritional values and assess food healthfulness.	*M* = 3.51, SD = 1.42	–	Weiss et al. (57)
Sex	Sex	What is your gender?	–	–	–
Age	Age	How old are you?	–	–	–
Body weight index (BMI)	Indicator of body weight category based on height and weight	How tall are you? (m) What’s your weight? (kg) BMI calculated as the ratio of weight to the square of height, categorizes individuals as underweight (BMI < 18.5), normal weight (BMI 18.5–23.9), or overweight (BMI ≥ 24).	–	–	–

#### 3.1.3 Data analysis

In this study, two ANCOVA models are employed to address research questions. In the first ANCOVA model, with FoPL types and color as fixed factors and HFI and FHL as covariates, FCC serves as the dependent variable, resulting in estimated marginal means (EMMs). In the second ANCOVA model, fixed factors and covariate variables were held constant, while the dependent variable was adjusted to HPC. Data analyses were performed using R.

### 3.2 Methods of Study 2

#### 3.2.1 Experimental design, sampling, and procedure

Study 2 was designed to complement Study 1 by shifting from a factorial comparison of design features to a configuration-level comparison of policy-relevant FoPL systems. In real-world FoPL policy, label format and color coding are often implemented as bundled configurations rather than as fully independent elements. Therefore, Study 2 focused on two representative policy examples: Australia’s HSR and the United Kingdom’s Multiple Traffic Light system. The HSR condition was represented by the Summary Label without Color Coding (SL), which summarizes the overall nutritional quality of a product through a single evaluative indicator. The Multiple Traffic Light condition was represented by the Nutrient-Specific Label with Color Coding (NSL), which presents nutrient-level information and uses color cues to indicate whether individual nutrient levels are relatively low, medium, or high. These two conditions were selected not to isolate the separate effects of label format and color coding again, but to compare two policy-relevant FoPL configurations in which these design features are commonly combined in practice.

To establish a baseline, Study 2 also included a No-FoPL control condition, while keeping all other package design elements identical across conditions. Thus, Study 2 employed a one-factor experimental design with three conditions: SL, NSL, and No-FoPL. This design allowed us to examine whether these two policy-relevant FoPL configurations were associated with consumers’ health perceptions and purchase intentions toward low-health products, and whether they showed different indirect pathway patterns involving nutrition accuracy and food health perception.

**FIGURE 5:**
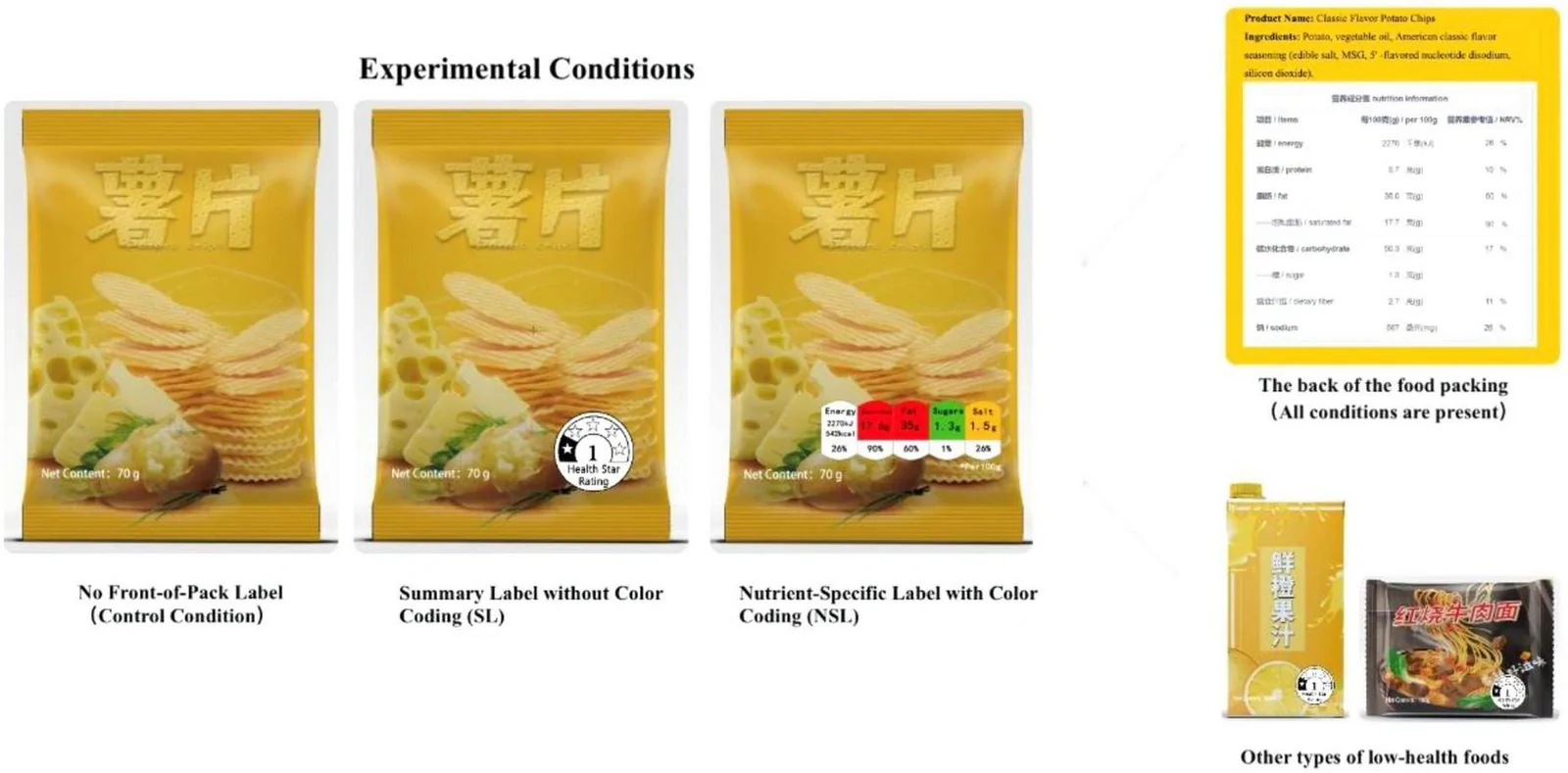
Experimental conditions in study 2.

Regarding the food types, Study 2 included a broader range of low-health packaged foods—instant meals, snacks, and beverages—to reduce the impact of personal food preferences and better assess the effects of FoPL. All items were rated 1 star under the Australian HSR system. The study aimed to test whether FoPL could lower health perceptions and purchase intentions for unhealthy foods (see Figure 5 for all experimental stimuli).

In the main experimental procedure, participants were randomly assigned to one of three conditions. In each condition, they were shown the front and back packaging of an instant noodle (instant food), a potato chip (snack), and an orange juice (beverage). After viewing each food item, participants immediately answered questions for the variables. After excluding 4 invalid responses identified through the attention check item, the final sample size was 138, which meets the criteria for sufficient statistical power and robust model estimation^7^ (see Table 3 for sample details).

**TABLE 3:** Sample description and random manipulation in Study 2.

Variables	Category	N	M/total score (SD)/percentage	Random test
Sex	Male	35	25.40%	χ^2^(2) = 2.898, *p* = 0.235
	Female	103	74.60%	
Age		138	21.36 (0.71)	*F*(2, 135) = 1.755, *p* = 0.177
BMI		138	20.39 (3.1)	*F*(2, 135) = 1.36, *p* = 0.259
Health food interest		138	4.45 (1.17)	*F*(2, 135) = 0.128, *p* = 0.88
Functional health literacy		138	3.8 (1.39)	*F*(2, 135) = 1.53, *p* = 0.219

#### 3.2.2 Measures

This study measured *Nutrition Accuracy, Food Health Perception*, *Intentions for Low-Healthy Food Purchases*, *Health Food Interest* (HFI), *Functional Health Literacy* (FHL) and demographic variables (see Table 4).

**TABLE 4:** Measurement details in Study 2.

Measures	Definition	Details	M/SD	α	References
Nutrition accuracy	Accuracy in identifying the levels of key nutrients in food products	Adapted from the Nutrient Understanding and Level-Estimation Accuracy test. Participants evaluated four indicators (fat, saturated fat, sugar, sodium) as “high,” “medium,” or “low” per UK traffic light label standards. Each correct response = 1 point, incorrect = 0. Three products × four indicators = total score out of 12.	*M* = 7.56, SD = 2.40	−	Roberto et al. (58)
Food health perception	Perceived healthiness of low-nutrition food categories	Adapted from the Health Motivation Scale. This [product category] is healthy. This [product category] is nutritious. This [product category] is good for me. (1 = strongly disagree to 7 = strongly agree) These items assess participants’ perceptions of each of the three food types: instant food, snack, and beverages.	instant food (M = 2.32 SD = 1.07), snacks (M = 2.24, SD = 0.98), and beverages (M = 3.7, SD = 1.64)	See Supplementary Appendix 1	Fernan et al. (31)
Intentions for low-healthy food purchases	Likelihood of purchasing low-health food products	I will buy this [product category]. Next time I am buying a [product category]. I will choose this product. I prefer this product to other [product category] options. (1 = strongly disagree to 7 = strongly agree) Lower scores for each food category indicate better outcomes for healthier decision-making.	Instant food (*M* = 2.97, SD = 1.4), snacks (*M* = 3.49, SD = 1.55), and beverages (*M* = 3.62, SD = 1.6)	Supplementary Appendix 1	Fernan et al. (31)
Health food interest (HFI)	Same as Study 1	Items: I pay a lot of attention to healthy foods. A healthy diet is very important to me. I pay close attention to the health benefits of food. I always eat what I want without worrying about the health of my diet (R). (This item was excluded from this study due to low factor loading). I inform myself very often about nutrition. (1 = strongly disagree to 5 = strongly agree) Higher scores indicate a greater interest in healthy eating.	*M* = 4.45, SD = 1.17	Supplementary Appendix 1	Steinhauser et al. (59)
Functional health literacy (FHL)	Same as Study 1	Same as Study 1	*M* = 3.51, SD = 1.42	–	Weiss et al. (57)
Sex	Sex	What is your gender?	–	–	–
Age	Age	How old are you?	–	–	–
Body weight index (BMI)	Indicator of body weight category based on height and weight	How tall are you? (m) What’s your weight? (kg) BMI calculated as the ratio of weight to the square of height, categorizes individuals as underweight (BMI < 18.5), normal weight (BMI 18.5–23.9), or overweight (BMI ≥ 24).	–	–	–

#### 3.2.3 Data analysis

Since *Food Health Perception* and *Intentions for Low-Healthy Food Purchases* consist of subdimensions, the analysis was conducted in two stages: validating the first-order measurement model and the second-order measurement model, and then testing the path relationships within the model. This two-step method allows antecedents to predict higher-order formative indicators, effectively avoiding the issue of indicator redundancy (60). To examine potential statistical indirect pathway patterns, this study used a PLS-SEM model due to its suitability for complex mediation models that test multiple variables and handle small-sample sensitivity (study 2: *N* = 138) (61, 62). Compared with covariance-based SEM, PLS-SEM is particularly suitable for exploratory and prediction-oriented research (63, 64). It has also been applied in recent healthy food consumption research to examine underlying psychological mechanisms (65). Accordingly, because the purpose of Study 2 was to identify the distinct psychological pathways through which each policy-relevant FoPL format operated relative to a no-label baseline, separate models were constructed for SL and NSL conditions (with the control group coded as 0 and FoPL-coded groups as 1) to explore potential difference paths. SmartPLS (version 4.0.8.7) was utilized for the analysis.

## 4 Results

### 4.1 Results of Study 1

Firstly, this study conducted random checks on the experiment using chi-square tests and ANOVA. The results indicated that age, gender, BMI, inspecting food packaging labels, HFI, and FHL were successfully distributed across different experimental groups (see Table 1). Furthermore, in order to assess the homogeneity of variances for both ANCOVA models, this study employed Levene’s Test on the dependent variables (FCC and HPC). The results revealed no significant deviations [FCC: *F*(3, 233) = 1.27, *p* = 0.17; HPC: *F*(3, 233) = 2.04, *p* = 0.1], thus satisfying the assumption of homogeneity for both models.

The first two-way ANCOVA was conducted to explore the influence of FoPL types and color on HPC while controlling for HFI and FHL (see Table 5). Regarding RQ1, the results of ANCOVA showed there is no significant main effect of FoPL types [*F*(1, 231) = 1.975, *p* = 0.16, partial η^2^ = 0.008] and color [*F*(1, 231) = 0.11, *p* = 0.73] on HPC. Moreover, there was a statistically significant two-way interaction between FoPL types and color on HPC [*F*(1, 231) = 11.08, *p* = 0.003, partial η^2^ = 0.046] while controlling for HFI and FHL (RQ2). Therefore, an analysis of simple main effects was conducted, with statistical significance receiving a Bonferroni adjustment and being accepted at the *p* < 0.05/2 = 0.025 level for color and *p* < 0.05/2 = 0.025 for FoPL types.

**TABLE 5:** Two-way ANCOVA results for health perceptional changes in Study 1.

Variables	*F*	*p*	η^2^
Health food interest	3.31	0.070	0.014
Functional health literacy	2.31	0.130	0.010
FoPL types	1.98	0.161	0.008
Color	0.11	0.738	ns
FoPL types × Color	11.08	< 0.001	0.046

Ns denotes that effect sizes were not calculated for statistically insignificant results.

**FIGURE 6:**
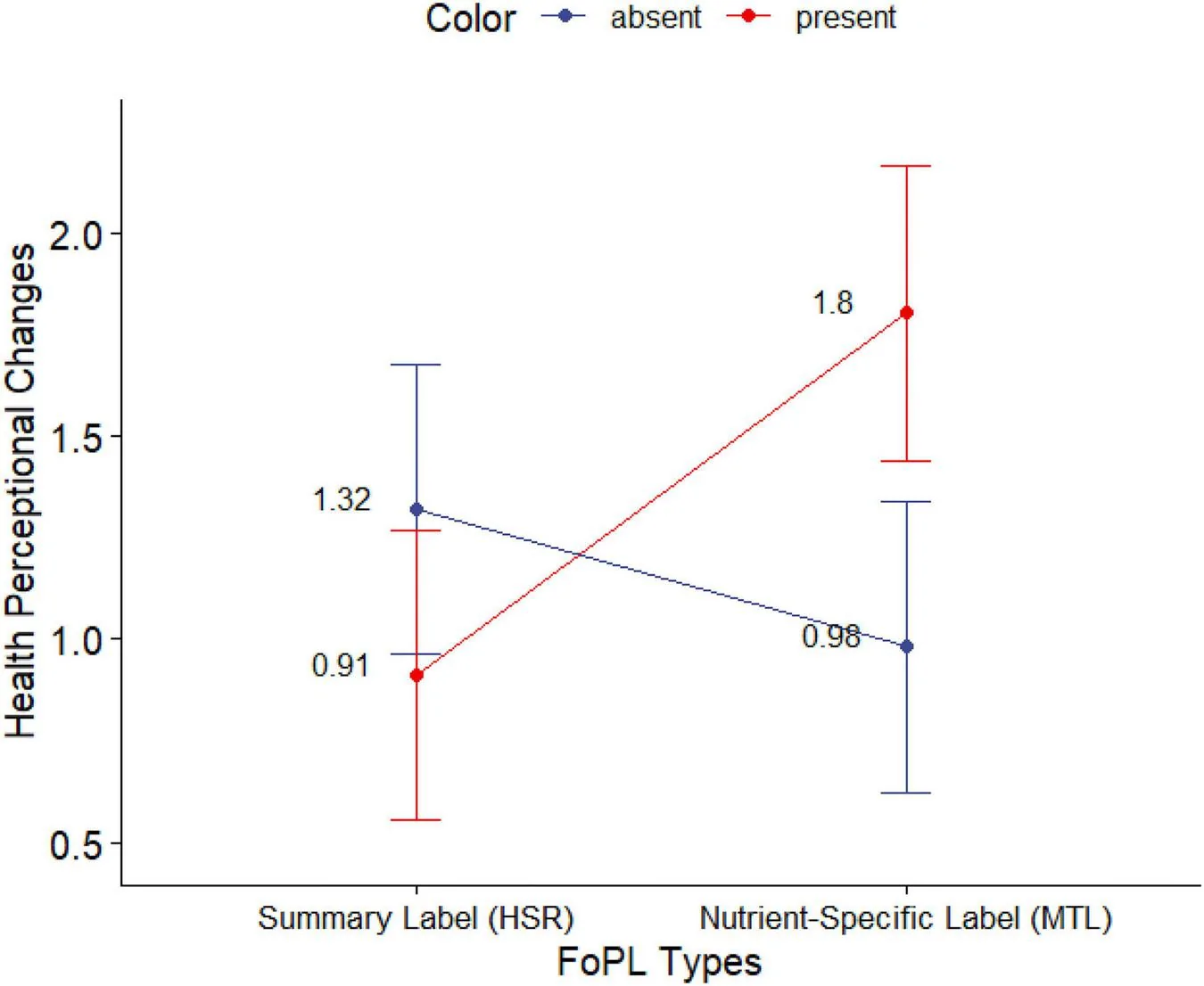
Interaction effect for Health Perceptional Changes displaying the estimated marginal means (EMMs) with 95% confidence interval error bars.

Figure 6 shows that the simple main effect of color was statistically significant in the nutrient-specific label (*p* = 0.001). More specifically, the nutrient-specific label with color (EMM = 1.8) is significantly higher than the nutrient-specific label without color (EMM = 0.98). Although there was no simple main effect of color that was statistically significant in the summary label, the summary label without color (EMM = 1.32) is significantly higher than the summary label with color (EMM = 0.91).

**FIGURE 7:**
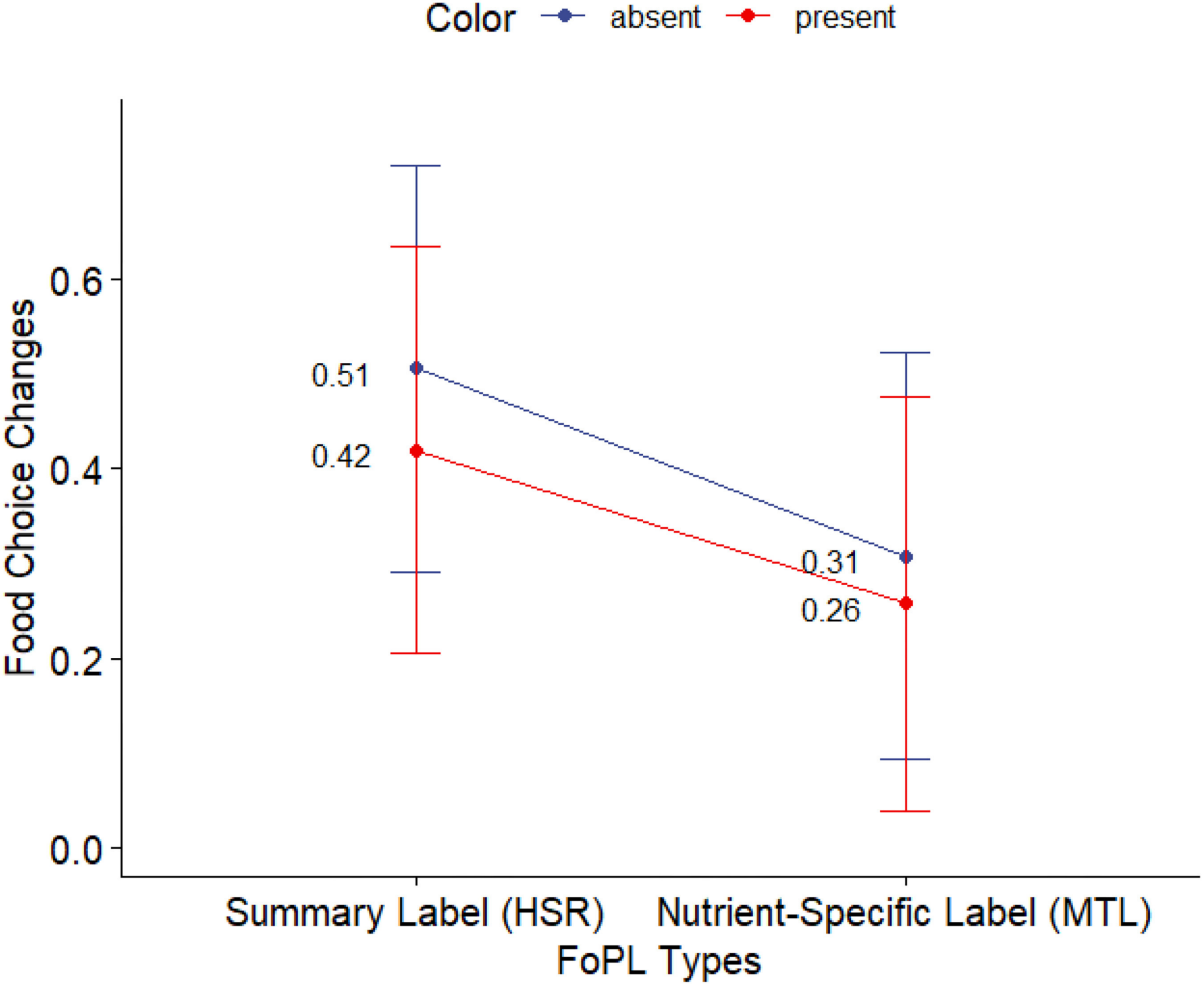
Food choice changes across all experimental conditions displaying the estimated marginal means (EMMs) with 95% confidence interval error bars.

The second two-way ANCOVA (Table 6) revealed that there were no significant main effects or interaction effects for FoPL types and Color on FCC (RQ2) (Figure 7 shows EMMs across all experimental conditions). The results also demonstrated that none of the covariates had a significant impact on FCC (all *p* > 0.05).

**TABLE 6:** Two-way ANCOVA results for food choice changes in Study 1.

	*F*	*p*	η^2^
Health food interest	1.77	0.185	0.008
Functional health literacy	2.78	0.097	0.012
FoPL types	3.26	0.072	0.014
Color	0.20	0.654	ns
FoPL types × Color	0.07	0.789	ns

Ns denotes that effect sizes were not calculated for statistically insignificant results.

### 4.2 Results of Study 2

Reliability and validity assessments were conducted for both PLS-SEM models. All first-order reflective and second-order formative constructs (food health perception and intentions for low-healthy food purchases) showed satisfactory reliability and validity, with all parameters meeting the recommended thresholds. Hence, both PLS-SEM models demonstrated satisfactory measurement quality. Full indicator details and estimation results, including the second-order measurement structure, are presented in Supplementary Appendix 1. The models showed meaningful explanatory power for intentions to purchase low-health foods, with R^2^ values of 0.504 in the SL model and 0.404 in the NSL model. The NSL model explained more variance in nutrition accuracy (*R*^2^ = 0.195) than the SL model (*R*^2^ = 0.059), which is consistent with the expectation that nutrient-specific labels support more detailed nutrient-level interpretation.

**FIGURE 8:**
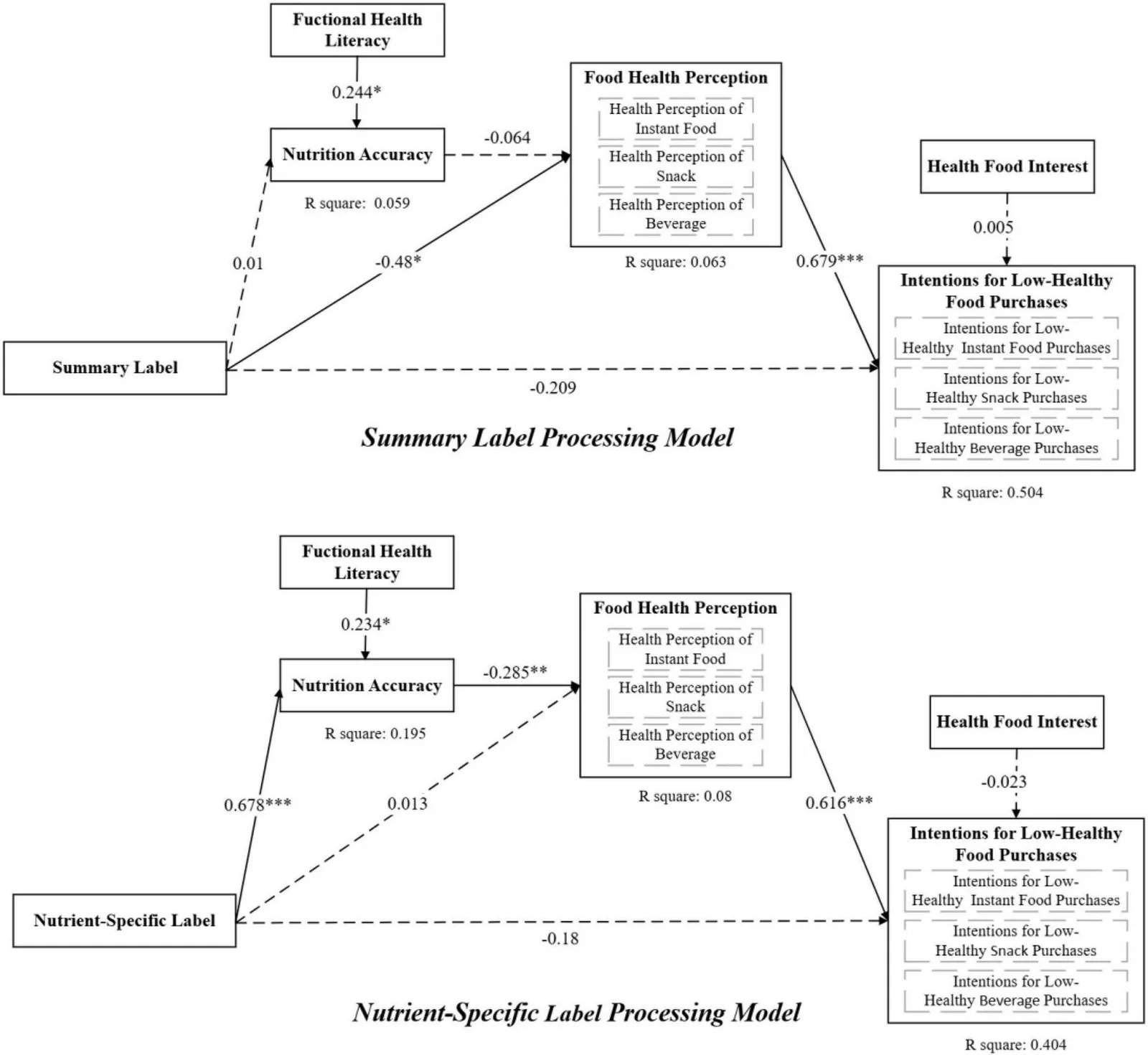
Results of SL and NSL models. *p < 0.05, **p < 0.01, and ***p < 0.001.

Regarding RQ3, PLS-SEM analyzed the hypothesized relationships among variables in the model, with Figure 8 illustrating the direct relationships between variables in both models and Table 7 displaying the indirect effect tests for the two models. In the SL model, mediation test results indicated that food health perception mediates the relationship between SL intervention and intentions for low-healthy food purchases (β = −0.326, *p* < 0.05).

**TABLE 7:** Indirect effects in the PLS-SEM mediation models.

Indirect path	Standardized coefficient	Standard deviation	*t*	*p*
SL				
SL - > nutrition accuracy - > food health perception	−0.001	0.028	0.002	0.998
SL - > Food health perception - > intentions for low-healthy food purchases	−0.326	0.148	2.198	0.028
Nutrition accuracy - > food health perception - > intentions for low-healthy food purchases	−0.044	0.075	0.583	0.560
SL - > nutrition accuracy - > food health perception - > intentions for low-healthy food purchases	−0.001	0.019	0.002	0.998
FHL- > nutrition accuracy - > food health perception	−0.016	0.031	0.509	0.611
FHL- > nutrition accuracy - > food health perception - > intentions for low-healthy food purchases	−0.011	0.021	0.517	0.606
NSL				
NSL - > nutrition accuracy - > food health perception	−0.193	0.09	2.136	0.033
NSL - > food health perception - > intentions for low-healthy food purchases	0.008	0.140	0.056	0.956
Nutrition Accuracy - > Food Health Perception - > Intentions for low-healthy food purchases	−0.176	0.067	2.624	0.009
NSL - > nutrition accuracy - > food health perception - > intentions for low-healthy food purchases	−0.119	0.059	2.018	0.044
FHL- > nutrition accuracy - > food health perception	−0.067	0.040	1.666	0.096
FHL- > nutrition accuracy - > food health perception - > intentions for low-healthy food purchases	−0.041	0.026	1.581	0.114

In the NSL model, mediation test results showed that nutrition accuracy mediates the relationship between the multiple traffic light label intervention and food health perception (β = −0.193, *p* < 0.05) and that food health perception mediates the relationship between nutrition accuracy and intentions for low-healthy food purchases (β = −0.176, *p* < 0.01). Overall, nutrition accuracy and food health perception significantly mediate the effect of the multiple traffic light label intervention on intentions for low-healthy food purchases (β = −0.119, *p* < 0.05).

## 5 General discussion

This study employed two experiments to examine how different FoPL formats and color-coding strategies influence consumers’ perceptions of food healthiness, healthy food choices, and intentions to purchase low-health foods, and to identify the psychological processing pathways through which FoPLs may promote healthier consumption. Overall, the findings suggest that summary labels and nutrient-specific labels showed relatively small differences in immediate behavioral change, but operated through different cognitive and motivational pathways. FoPLs should therefore not be understood only as information-provision tools, but also as interpretive cues that shape how consumers simplify, evaluate, and use nutrition information.

### 5.1 FoPLs shape perception before choice

Study 1 found that the four FoPL conditions, regardless of type or color coding, generally improved consumers’ health perceptions compared with the traditional NFP baseline. This finding is consistent with prior research suggesting that FoPLs can help consumers evaluate the nutritional quality of food products more easily and reduce reliance on more complex back-of-package nutrition information (14, 66, 67). However, the interpretation of this finding should be limited to health perception rather than extended to immediate behavioral change. Because Study 1 did not identify significant main effects or interaction effects of FoPL type and color coding on food choice changes, the results should not be interpreted as evidence that different FoPL designs produced significantly different behavioral choice outcomes. Rather, they suggest that FoPLs may have clearer and more immediate effects on health-related perceptions than on actual food choice changes in a one-time experimental task.

### 5.2 Color coding needs nutritional context

Study 1 further found that FoPL type and color-coding strategy had a significant interaction effect on changes in perceived healthiness. Specifically, nutrient-specific labels combined with color coding produced the strongest improvement in health perception. This interaction result suggest that color coding should not be understood as a universally effective visual feature, but as an interpretive cue whose value depends on the label information structure. Nutrient-specific labels provide multiple pieces of nutrient-level information, and color cues can help consumers attach evaluative meaning to these details, such as whether sugar, sodium, or saturated fat levels are relatively low, medium, or high. This finding is consistent with the logic of real-world FoPL systems, such as Multiple Traffic Light-style labels, in which colors help consumers interpret specific nutrient levels.

In contrast, summary labels simplify nutrition information into an overall evaluation. In Study 1, the color strategy for summary labels provided limited incremental information because the star rating itself had already communicated an overall health evaluation. Although the summary label also used multiple colors in the color-present condition, these colors were attached to an overall star rating rather than to specific nutrient dimensions. This may explain why the color-coded nutrient-specific label significantly outperformed the color-coded summary label in improving perceived healthiness. Consistent with color-in-context theory, color effects depend not only on color itself, but also on the interpretive context in which color is embedded (34, 68). In the present study, color coding was more effective when it complemented nutrient-specific information than when it merely reinforced an already simplified summary evaluation.

### 5.3 How FoPLs work: simplicity and detail as different pathways

Study 2 further clarified the indirect models through which two policy-relevant FoPL configurations (summary label: HSR and color-coded nutrient-specific label: Multiple Traffic Light) influence intentions to purchase low-health foods. Specifically, summary labels mainly reduced consumers’ intentions to purchase low-health foods by shaping food health perception, whereas nutrient-specific labels with color coding operated through a sequential pathway involving nutrition accuracy and food health perception. This finding moves FoPL research beyond the question of which label is more effective and toward a more mechanism-oriented question of how different FoPL formats work.

The HSR summary-label condition showed a significant indirect association with lower intentions to purchase low-health foods through food health perception. From the perspective of heuristic–systematic processing theory, summary labels are more likely to function as heuristic cues because they condense complex nutrition information into a single evaluative signal (46). This allows consumers to form quick health judgments with relatively low cognitive effort. This pathway is consistent with prior research suggesting that summary labels can support rapid product evaluation by simplifying nutrition information (19, 26, 69). It also corresponds to the design logic of HSR-style systems, which translate multiple nutrient attributes into an overall star rating to facilitate product-level comparison. However, because summary labels compress detailed nutrition information, they may shape overall health perception without necessarily improving consumers’ nutrient-level understanding.

By contrast, the MTL color-coded nutrient-specific condition showed a significant sequential indirect association involving nutrition accuracy and food health perception. Nutrient-specific labels provide more detailed information about individual nutrients. When combined with color coding, unfavorable nutrient levels become more visible and easier to interpret. This aligns with prior research showing that nutrient-specific labels can support more deliberate evaluation of nutrients of concern, such as sugar, sodium, and saturated fat (11, 16, 23). This Study 2 pattern is consistent with the interaction observed in Study 1: color coding appears most useful when it is embedded in a nutrient-specific information structure, where it helps translate detailed nutrient values into evaluative meanings. In this sense, the difference between the HSR summary label and the MTL color-coded nutrient-specific label reflects a trade-off between simplicity and diagnostic detail.

Taken together, they suggest that FoPLs may first influence more proximal cognitive outcomes, such as food health perception and nutrition accuracy, and then shape motivational outcomes, such as intentions to purchase low-health foods, before these effects become visible in more distal immediate choice behavior. Specifically, color-coded nutrient-specific labels should be understood as a trade-off between simplicity and diagnostic detail. Summary labels may be more suitable for supporting quick, low-effort product comparison, whereas nutrient-specific labels may be more suitable for making specific nutrient risks visible and supporting consumers’ understanding of why a product is less healthy. Importantly, the effect-size results indicate that FoPLs in this study operated mainly through proximal cognitive mechanisms rather than through large direct effects on behavioral outcomes. In Study 2, the models explained a meaningful proportion of variance in low-health food purchase intentions, with R^2^ values of 0.504 in the SL model and 0.404 in the NSL model, and food health perception showed a large effect on purchase intentions in both models. This suggests that the practical value of FoPLs lies less in producing immediate behavioral change after a single exposure and more in shaping the cognitive conditions under which healthier decisions become more likely.

The findings also clarify the role of individual differences. Contrary to some prior research (7), Study 1 found no significant direct effect of functional health literacy or health food interest on food choice changes. However, Study 2 revealed that functional health literacy was associated with nutrition accuracy, which was an important cognitive mediator in the nutrient-specific label pathway. Thus, this study identifies the role of nutrition accuracy in linking health literacy with health-related purchase intentions. This suggests that functional health literacy may not directly change immediate food choice, but it can help consumers interpret detailed nutrient information when the label requires more analytical processing. Unexpectedly, health food interest did not influence purchase intentions. One possible explanation is that healthier food decisions involve multiple competing factors, such as personal preferences, taste, and habitual consumption (40, 70).

### 5.4 Practical implications

The findings provide implications for policymakers, food companies, and retailers. For policymakers, the results suggest that FoPLs can serve as useful interpretive tools to complement traditional NFPs, particularly by making nutrition information easier to understand and evaluate. Although the evidence for immediate behavioral change remains limited, the practical value of FoPLs may lie mainly in improving the interpretability of nutrition information and creating more favorable cognitive conditions for healthier decisions. Second, when employing different strategies for FoPL, it is crucial to focus on key factors for processing the strategy information. For example, nutrient-specific labels should emphasize a clear explanation of specific nutritional factors, while summary labels should focus on how to quickly perceive overall health levels and guide understanding of detailed nutritional information. Currently, this study suggests that when FoPL policies require a single color, a numerical summary or summary label (e.g., HSR) can be used. When FoPL policies allow for multiple colors, nutrient-specific labels with color explanations (e.g., MTL) can be adopted. Additionally, alongside the implementation of FoPL policies, efforts should also focus on enhancing functional health literacy related to nutrition information intake and nutritional calculations. These complementary efforts may help consumers make better use of FoPL information and support more informed dietary decisions.

For food companies and retailers, the findings suggest that packaging and point-of-sale communication should be matched to the intended communication goal. When the goal is to provide a simple and intuitive overall health signal, summary labels may be more useful. When the goal is to communicate nutritional strengths or weaknesses, color-coded nutrient-specific labels may be more appropriate. Because nutrient-specific color-coded labels make unfavorable nutrient profiles more visible, they may encourage companies to reduce sugar, sodium, or saturated fat and improve their front-of-package presentation. Retailers can also integrate FoPLs into product comparison cues and healthier-choice prompts. Especially for color-coded FoPLs, placing products side by side may help consumers interpret color meanings through comparison, thereby reinforcing label information in the shopping environment rather than leaving labels to work in isolation.

### 5.5 Limitations and future research

This study has several limitations. First, both studies relied on convenience samples of university students, which limits the generalizability of the findings. Although this sample was useful for testing initial mechanisms among consumers with limited prior exposure to FoPLs, university students may differ from the broader population in age, income, food purchasing responsibility, nutrition knowledge, and shopping habits. Future research should examine more diverse and representative consumer groups, including older adults, parents, consumers with lower nutrition literacy, and individuals affected by obesity or nutrition-related chronic diseases. Second, Study 2 measured intentions to purchase low-health foods rather than actual purchasing behavior. Although purchase intention is an important motivational outcome, it does not necessarily translate into real-world purchasing behavior. Actual purchases may be influenced by price, promotions, brand familiarity, taste, and shopping context (3, 40, 71). Future studies should therefore test FoPL effects in more realistic purchasing environments, such as online supermarkets, simulated stores, using actual purchase data. Third, although the PLS-SEM results showed significant indirect associations, they cannot establish temporal ordering or causal mechanisms of nutrition accuracy to food health perception and purchase intention. Future studies could use longitudinal, sequential, or process-tracing designs, such as multi-stage experiments or response-time measures to examine how consumers process FoPL information over time. Finally, although this study examined the roles and mechanisms of summary labels and nutrient-specific labels (e.g., HSR and MTL), other FoPL systems, such as warning labels, Nutri-Score, reference intake labels, or hybrid labels, may have different effects on information processing and health-related decision-making. Future research should compare a wider range of FoPL systems and examine whether their effects vary across product categories, consumer groups, and levels of product healthfulness.

## Data Availability

The raw data supporting the conclusions of this article will be made available by the authors, without undue reservation.

## Footnotes

1 The MTL system employs color-coded labels on the front of packaging to indicate nutrient levels—green for low, orange for moderate, and red for high—based on recommended daily intake values.

2 The HSR system provides an overall nutrition rating, summarizing health quality through a star rating.

3
www.wjx.com

4 This study selected yogurt as the food product due to its widespread consumption and variable health attributes associated with fat and sugar content, enabling it to be categorized as either healthy or unhealthy (72). Unlike foods typically perceived as unhealthy (e.g., chips), yogurt is generally seen as healthy, which may lead to misconceptions about its nutritional quality.

5 Health quality based on the HSR standard (1–2 stars = low, 2.5–3.5 = medium, 4–5 = high), each with distinct packaging.

6 A priori power analysis for ANCOVA indicated that a minimum sample size of 158 was required (Cohen’s *f* = 0.25, power = 0.8, α = 0.05), confirming that the obtained sample was adequate.

7 Partial Least Squares Structural Equation Modeling (PLS-SEM) was employed for data analysis. According to the 10-times rule (73), the minimum required sample size for this model is 70. To further verify sample adequacy, a power analysis based on multiple regression principles (63) was conducted. The required sample size to detect R^2^ values ranging from 0.10 to 0.75 (α = 0.05, power = 0.80, seven predictors) is approximately between 61 and 118. The final samples of 86 and 93 in the two models therefore meet these criteria.
