# Supplementary material for: Big picture vs. small details: exploring how front-of-package labels influence healthy food consumption and cognitive processing
Source: Front Nutr. 2026 Aug 14;13:1881270. doi: 10.3389/fnut.2026.1881270 (PMC13521866; doi:10.3389/fnut.2026.1881270)
Supplement: Supplementary file 1 [file Supplementary_file_1.docx]

**Appendix A**

To assess the validity of the first-order measurement model, this study examined both convergent and discriminant validity as well as the reliability of the constructs. All first-order factors were reflective in nature. Table A-1 presents the correlation matrix for the constructs and each indicator within the first two first-order models. Factor loadings (FL) for each item exceeded the threshold of 0.6, with Cronbach’s alpha and composite reliability (CR) values surpassing the standard of 0.7, and average variance extracted (AVE) values above 0.5 (Hair et al., 2019; Iqbal et al., 2022). Thus, the data demonstrated reliability and validity across both constructs.

| **Table A-1. Bivariate Correlations Among the First-order Factors** | | | | | | | | | | | | | |
| --- | --- | --- | --- | --- | --- | --- | --- | --- | --- | --- | --- | --- | --- |
| **SL** | | | | | | | | | | | | | |
|  | **α** | **CR** | **AVE** | **FL** | **1** | **2** | **3** | **4** | **5** | **6** | **7** | **8** | **9** |
| 1. Health Perception of Instant Food | .886 | .891 | .815 | .868-.929 |  |  |  |  |  |  |  |  |  |
| 2. Health Perception of Snack | .871 | .815 | .795 | .866-.92 | .58** |  |  |  |  |  |  |  |  |
| 3. Health Perception of Beverage | .95 | .965 | .909 | .926-.96 | .50** | .38** |  |  |  |  |  |  |  |
| 4. Intentions for Instant Food Purchases | .902 | .889 | .837 | .899-.935 | .58** | .31** | .45** |  |  |  |  |  |  |
| 5. Intentions for Snack Purchases | .911 | .872 | .849 | .911-.936 | .37** | .60** | .32** | 2.1* |  |  |  |  |  |
| 6. Intentions for Beverage Purchases | .94 | .95 | .893 | .94-.948 | .30** | .37** | .61** | .28** | .53** |  |  |  |  |
| 7.Health Food Interest | .881 | .898 | .599 | .795-.9 | -.13 | -.27* | -.18 | .02 | -.21* | -.11 |  |  |  |
| 8.Fuctional Health Literacy | | | |  | -.18 | -.18 | -.11 | -.13 | -.03 | -.12 | -.04 |  |  |
| 9.Nutrition Accuracy | | |  |  | -.21* | -0.13 | .15 | -.15 | .13 | .17 | .04 | .24* |  |
| 10.Health Star Rating | | |  |  | -.23* | -0.1 | -.25* | -.22* | -.16 | -.23* | -.04 | .15 | .04 |
| **NSL** | | | | | | | | | | | | | |
| 1.Health Perception of Instant Food | .889 | .891 | .819 | .876-.937 |  |  |  |  |  |  |  |  |  |
| 2.Health Perception of Snack | .804 | .815 | .722 | .761-.926 | .51** |  |  |  |  |  |  |  |  |
| 3.Health Perception of Beverage | .964 | .965 | .933 | .928-.963 | .27** | .42** |  |  |  |  |  |  |  |
| 4.Intentions for Instant Food Purchases | .915 | .915 | .854 | .96-0.97 | .58** | .20* | .30** |  |  |  |  |  |  |
| 5.Intentions for Snack Purchases | .931 | .931 | .88 | .912-.943 | .34** | .51** | .33** | .32** |  |  |  |  |  |
| 6.Intentions for Beverage Purchases | .945 | .946 | .9 | .94-.954 | .09 | .30** | .60** | .15 | .47** |  |  |  |  |
| 7.Health Food Interest | .776 | .783 | .599 | .63-.868 | -.06 | -.17 | -.12 | -.03 | -.13 | -.04 |  |  |  |
| 8.Fuctional Health Literacy | | | |  | -.05 | -.01 | -.15 | -.11 | -.01 | -.12 | .14 |  |  |
| 9.Nutrition Accuracy | | |  |  | -.33** | -.21* | -.12 | -.22* | -.11 | -.07 | 0.04 | .29** |  |
| 10.Multiple Traffic Light | | |  |  | -.10 | -.09 | -.06 | -.06 | -.16 | -.10 | -.01 | .17 | .37** |
| *Note*. **p* <.05, ***p* <.01. FoPL condition = 1, control condition = 0. | | | | | | | | | | | | | |

Both models include two formative second-order factors: intentions for low-healthy food purchases and food health perception. These second-order factors, constructed from the first-order reflective factors, allow this study to examine higher-level psychological constructs within the model. Table A-2 presents the bootstrap analysis estimates based on 5,000 samples. All path coefficients are substantial and significant at *p* < .001, indicating strong construct validity for the formative factors.

| **Table A-2. Upward Dimension Effects for Food Health Perception and Intentions for Low-Healthy Food Purchases** | | | | | |
| --- | --- | --- | --- | --- | --- |
| **SL** | | | | | |
| **Relationship** | **Original β** | **Mean β** | **STDEV** | **t** | ***p*** |
| Health Perception of Instant Food -> Food Health Perception | 0.426 | 0.426 | 0.029 | 14.816 | <.001 |
| Health Perception of Snack -> Food Health Perception | 0.387 | 0.386 | 0.025 | 15.621 | <.001 |
| Health Perception of Beverage -> Food Health Perception | 0.418 | 0.417 | 0.028 | 14.904 | <.001 |
| Intentions for Instant Food Purchases-> Intentions for low-healthy food purchases | 0.343 | 0.324 | 0.083 | 4.138 | <.001 |
| Intentions for Snack Purchases-> Intentions for low-healthy food purchases | 0.474 | 0.475 | 0.043 | 11.117 | <.001 |
| Intentions for Beverage Purchases-> Intentions for low-healthy food purchases | 0.492 | 0.49 | 0.044 | 11.076 | <.001 |
| **NSL** | | | | | |
| Health Perception of Instant Food -> Food Health Perception | 0.43 | 0.425 | 0.037 | 11.497 | <.001 |
| Health Perception of Snack -> Food Health Perception | 0.414 | 0.415 | 0.032 | 12.817 | <.001 |
| Health Perception of Beverage -> Food Health Perception | 0.447 | 0.446 | 0.041 | 11.019 | <.001 |
| Intentions for Instant Food Purchases-> Intentions for low-healthy food purchases | 0.362 | 0.344 | 0.084 | 4.321 | <.001 |
| Intentions for Snack Purchases-> Intentions for low-healthy food purchases | 0.514 | 0.513 | 0.041 | 12.449 | <.001 |
| Intentions for Beverage Purchases-> Intentions for low-healthy food purchases | 0.46 | 0.459 | 0.051 | 9.086 | <.001 |

Study 2 assessed discriminant validity for causal association factors at the highest level using the Heterotrait-Monotrait ratio test. **Table A-3** shows that all values meet this criterion (the ratio < 1.00), indicating good discriminant validity among constructs (Henseler et al., 2016).

| **Table A-3. Heterotrait-Monotrait Ratios** | | | | | |
| --- | --- | --- | --- | --- | --- |
| **SL** | | | | | |
|  | **1** | **2** | **3** | **4** | **5** |
| 1.Food Health Perception |  |  |  |  |  |
| 2.Intentions for low-healthy food purchases | 0.807 |  |  |  |  |
| 3.Nutrition Accuracy | 0.215 | 0.212 |  |  |  |
| 4.Health Star Rating | 0.257 | 0.295 | 0.037 |  |  |
| 5.Health Food Interest | 0.273 | 0.208 | 0.046 | 0.051 |  |
| 6.Functional Health Literacy | 0.203 | 0.13 | 0.244 | 0.148 | 0.063 |
| **NSL** | | | | | |
|  | 1 | 2 | 3 | 4 | 5 |
| 1.Food Health Perception |  |  |  |  |  |
| 2.Intentions for low-healthy food purchases | 0.732 |  |  |  |  |
| 3.Nutrition Accuracy | 0.303 | 0.197 |  |  |  |
| 4.Multiple Traffic Light | 0.108 | 0.197 | 0.376 |  |  |
| 5.Health Food Interest | 0.225 | 0.154 | 0.058 | 0.07 |  |
| 6.Functional Health Literacy | 0.143 | 0.118 | 0.291 | 0.168 | 0.169 |

Although PLS-SEM differs from covariance-based SEM (CB-SEM) and is generally considered more explanation- and prediction-oriented than global-fit-oriented, accordingly, the SRMR and NFI values were reported as supplementary transparency indicators rather than as the primary basis for evaluating the adequacy of the PLS-SEM models (Hair et al., 2022). The estimated Standardized Root Mean Square Residual (SRMR) values were .125 for the SL model and .102 for the NSL model, while the Normed Fit Index (NFI) values were .327 and .290, respectively. These results suggest that the approximate global fit of the two models was limited. However, the Study 2 models included higher-order formative constructs and were designed primarily to examine explanatory mechanisms rather than to establish a covariance-based model with strong global fit. This treatment is consistent with methodological discussions suggesting that PLS-SEM model evaluation should not rely exclusively on global goodness-of-fit criteria, particularly when models include composite or formative constructs (Hair et al., 2022; Henseler et al., 2016). Therefore, the main interpretation of the PLS-SEM results focused on measurement quality, explanatory power, effect sizes, predictive assessment, and bootstrapped indirect effects.

**References**

Hair, J., Hult, G. T. M., Ringle, C. M., Sarstedt, M. (2016). A Primer on Partial Least Squares Structural Equation Modeling (PLS-SEM). United States: SAGE Publications.

Hair, J. F., Risher, J. J., Sarstedt, M., & Ringle, C. M. (2019). When to use and how to report the results of PLS-SEM. European Business Review, 31(1), 2–24. https://doi.org/10.1108/EBR-11-2018-0203

Henseler, J., Ringle, C. M., & Sarstedt, M. (2015). A new criterion for assessing discriminant validity in variance-based structural equation modeling. Journal of the Academy of Marketing Science, 43(1), 115–135. <https://doi.org/10.1007/s11747-014-0403-8>

Henseler, J., Hubona, G., & Ray, P. A. (2016). Using PLS path modeling in new technology research: updated guidelines. Industrial management & data systems, 116(1), 2-20.

Iqbal, J., Asghar, M. Z., Ashraf, M. A., & Rafiq, M. (2022). Social Media Networking Sites Usage and Depression Among University Students During the COVID-19 Pandemic: The Mediating Roles of Social Anxiety and Loneliness. Social Media + Society, 8(3), 20563051221107633. https://doi.org/10.1177/20563051221107633
